# Advantages and Limitations of Fluorescence Lifetime Measurements Using Single-Photon Avalanche Diode (SPAD) Array Detector: A Comprehensive Theoretical and Experimental Study

**DOI:** 10.3390/s22103822

**Published:** 2022-05-18

**Authors:** Alexander Netaev, Nicolas Schierbaum, Karsten Seidl

**Affiliations:** 1 Fraunhofer Institute for Microelectronic Circuits and Systems, 47057 Duisburg, Germany; nicolas.schierbaum@ims.fraunhofer.de (N.S.); karsten.seidl@uni-due.de (K.S.); 2 Department of Electronic Components and Circuits, University of Duisburg-Essen, 47057 Duisburg, Germany

**Keywords:** SPAD, fluorescence lifetime, pile-up, Monte Carlo, detector array

## Abstract

Fast fluorescence lifetime (FL) determination is a major factor for studying dynamic processes. To achieve a required precision and accuracy a certain number of photon counts must be detected. FL methods based on single-photon counting have strongly limited count rates because of the detector’s pile-up issue and are suffering from long measurement times in the order of tens of seconds. Here, we present an experimental and Monte Carlo simulation-based study of how this limitation can be overcome using array detectors based on single-photon avalanche diodes (SPADs). We investigated the maximum count rate per pixel to determine FL with a certain precision and accuracy before pile-up occurs. Based on that, we derived an analytical expression to calculate the total measurement time which is proportional to the FL and inversely proportional to the number of pixels. However, a higher number of pixels drastically increases data rate. This can be counteracted by lowering the time resolution. We found that even with a time resolution of four times the FL, an accuracy of 10% can be achieved. Taken all together, FLs between 10 ns and 3 ns can be determined with a 300-pixel SPAD array detector with a measurement time and data rate less than 1 µs and 700 Mbit/s, respectively. This shows the enormous potential of SPAD array detector for high-speed applications requiring continuous data read out.

## 1. Introduction

Measuring the fluorescence lifetime (FL) has gained rising interest in the field of biomedicine with numerous applications such as studying molecular interactions [1,2], monitoring environmental parameters (pH [3,4,5], temperature [6] and ion concentration [7]), and for multi-parametric bioassays [8]. FL is the average time a fluorophore remains in the excited state prior returning to ground state by emitting a fluorescence photon [9]. Compared with intensity-based fluorescence measurement, FL as an intrinsic material parameter has the advantage of being independent of fluorophore concentration and thus of intensity [10]. In addition, FL is very attractive for multiplexing in bioassays since it allows to distinguish between fluorophores with overlapping spectral properties based on their characteristic FL [11].

There are two main approaches to measure FL, either in time-domain or in frequency-domain [12]. FL measurements in the time-domain are advantageous over frequency-domain when, for instance, studying complex multi-exponential decays of the fluorescence signal [9]. In time-domain methods, e.g., time-correlated single-photon counting (TCSPC), the sample is periodically excited by a short light-pulse (e.g., laser) and the arrival times of emitted fluorescence photons at a single-photon sensitive detector are recorded to reconstruct the temporal decay of the fluorescence intensity characterized by the FL [12]. However, precision and accuracy of FL determination strongly depends on the number of detected photons [13]. Since in conventional TCSPC the probability of photon detection is kept below one photon per measurement window to avoid an underestimation of the FL by the pile-up effect as a result of detector’s dead time (typically tens of ns) [14], TSCPC generally requires long measurement times. This is extremely crucial when it comes to highly dynamic applications such as flow cytometry [15] or high-speed imaging [16].

To overcome this limitation in recent years different approaches have been proposed to reduce the detector’s dead time such as hybrid photodetectors, i.e., vacuum tube-based electronic acceleration combined with avalanche diodes with dead times below 1 ns [17,18], or software-based dead-time corrections [19]. One further promising approach is using pixelated array detectors based on single-photon avalanche diodes (SPADs) instead of conventional photomultiplier tubes (PMT) with “one pixel” (i.e., one photosensitive area) [19,20,21]. SPAD array detectors have the advantage that during one measurement window more than one photon can be detected. Hence, a shorter measurement time is needed to detect the same number of photons than using a “one-pixel” detector [22]. In this regard the question of how the precision and accuracy of FL determination correlates with total photon counts and the rate at which they are detected arises. We determined the maximum count rate before pile-up negatively impacts the accuracy and calculated the total measurement time in dependency of different FLs and number of pixels. We show that 30 and 300 pixels are sufficient to determine FLs <10 ns within a measurement time less than 10 µs and less than 1 µs, respectively. To enable continuous FL measurement, the data rate of SPAD array detector must be kept below system limitations, e.g., by the speed of the data interface. The data rate is mainly determined by the time resolution. We show that a time resolution of four times the FL is sufficient to determine the FL with an accuracy of 10%. For FLs ≥ 3 ns with 300 pixels, this leads to data rates of less than 700 Mbit/s, which can be transferred with most modern circuits.

## 2. Background

**Figure 1:**
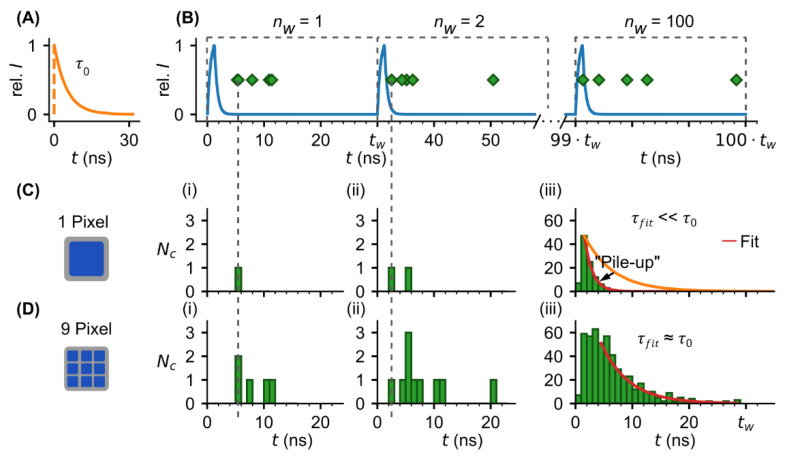
TCSPC-based FL measurements using SPAD array detector. (**A**) “Ideal” fluorescence signal (intensity vs. time) of a fluorophore with a single-exponential decay characterized by its lifetime $\tau_{0}$. (**B**) Lifetime measured by TCSPC. Fluorophores are excited with a short laser pulse (blue curve) and the arrival times of emitted fluorescence photons (green markers) are detected by a single-photon detector, e.g., PMT or SPAD array detector. To obtain a sufficient number of arrival times, laser excitation and photon counting are repeated for $n_{w}$ - times with a defined measurement window duration $t_{w}$. All arrival times are stored in a histogram allowing to extract the lifetime by exponential fit (**Ciii**,**Diii**). The count rate during each measurement window depends on the number of individual photosensitive areas or pixels of the detector (**C**,**D**). With “one-pixel” single-photon detector, only the first photon per measurement window can be detected (**Ci**,**Cii**). In case of high photon rates (photons per measurement window), this “first-photon” issue leads to a pile-up of the histogram and an underestimation (lower accuracy) of the lifetime as indicated by difference between the fit (red curve) and ideal fluorescence signal (orange curve from (**A**)) (**Ciii**). To avoid pile-up, the photon rate should be smaller than the number of pixels. With “pixelated” SPAD array detector higher photon rates can be permitted (**D**, here, pixels=9 > photon rate $<N_{c}{>}_{w}=5$ ) resulting in higher total counts (**Di, Dii**) and a more accurate estimation of the lifetime (**Diii**) compared with one pixel (**Ciii**) after the same number of measurement windows (i.e., same total measurement time).

In TCSPC, due to the Poissonian nature of photon statistics and experimental noise, the intrinsic FL of a fluorophore (Figure 1A) can only be determined with a certain accuracy. To provide a fundamental understanding of the measurement principle in TCSPC and how the accuracy is affected by the count rate, we illustrated the determination of FL at relatively high photon rate for a “one-pixel” single-photon detector such as a PMT [23] (Figure 1C) and a “pixelated” SPAD array detector (Figure 1D).

In TCPSC, the temporal decay of the fluorescence intensity is obtained by detecting arrival times of single-photon events emitted by a fluorophore after short-laser pulse excitation and sorting the events in a histogram from which the FL is determined by curve fitting (Figure 1C,D).

With a “one-pixel” single-photon detector only one photon per laser excitation cycle can be detected (Figure 1C). One cycle is declared a measurement window $n_{w}$ with the duration $t_{w}$ throughout the study.

This photon count limitation makes TCSPC a cumbersome method because the count rate must be adjusted by tuning the laser power so that the probability to detect a photon is below one photon per measurement window. If count rates are higher than the number of pixels, photons are missed by the detector. In this case, only the fastest photons are detected (Figure 1(Ci,Cii)). This so-called “first photon” problem causes a pile-up of the histogram (Figure 1(Ciii)), resulting in a lower accuracy of FL acquisition. This problem is well-known from literature [20,24]. Several methods have been developed to back-calculate the pile-up [19,25]. However, the use of these methods is limited, e.g., for multiexponential decays with multiple fluorophores, information on the fractions in which the fluorophores are present is required.

With a “pixelated” SPAD array detector, the higher number of pixels allows for higher photon rates; so in the example shown FL detection is less affected by pile-up than in a “single-pixel” single-photon detector (Figure 1(Diii)). With such a SPAD array detector, less time is needed to collect a sufficient number of photons. In simple terms, if one pixel is dead due to photon hit the second one is still active to catch an additional photon. However, it remains to be investigated how the pile-up is related to the number of pixels and what measurement speed can be achieved with a given number of pixels.

## 3. Materials and Methods

### 3.1. Experimental Setup

**Figure 2:**
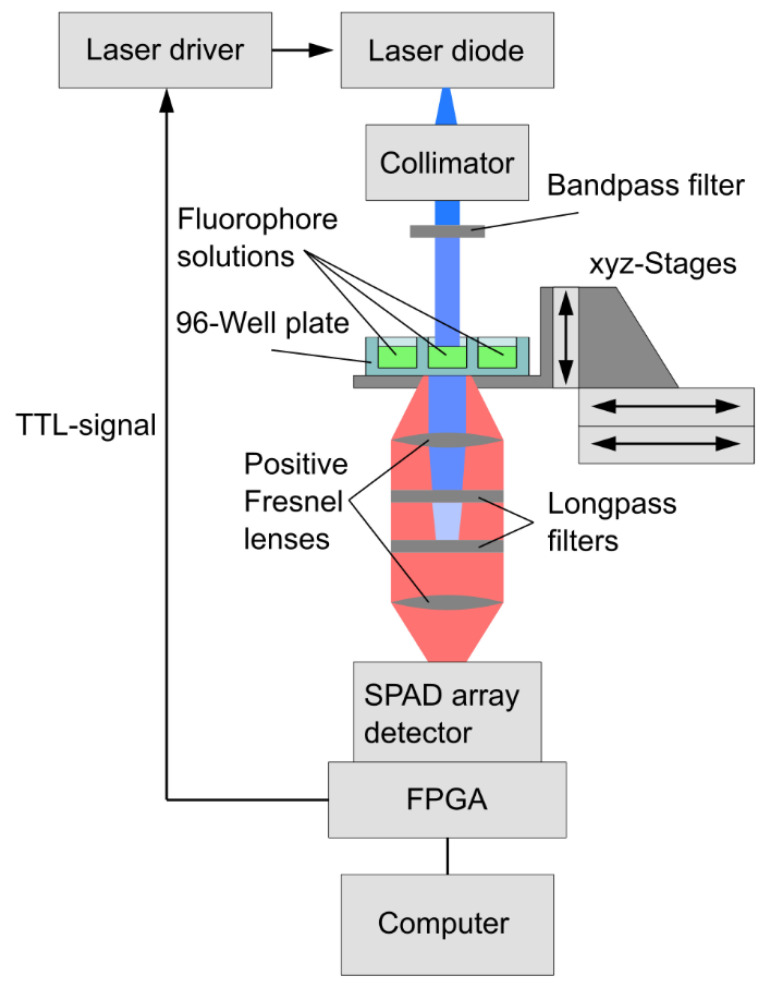
Schematic of the experimental FL setup. The fluorophore is excited by a collimated pulsed laser diode (λ=450 nm), filtered by a bandpass filter. The emitted fluorescence is collected and focused onto the SPAD array detector by two positive Fresnel lenses. Long-pass filters were used to filter out residual signal from the pulsed laser diode. A FPGA board was used to control trigger signals for laser and SPADs and to process the data from the SPAD array detector.

FL measurements were carried out using a custom-built setup based on a 192 × 2 pixel CMOS SPAD array detector, which was developed by the Fraunhofer IMS and has a time resolution of 312.5 ps [26] (cf. Figure 2).

**Figure 3:**
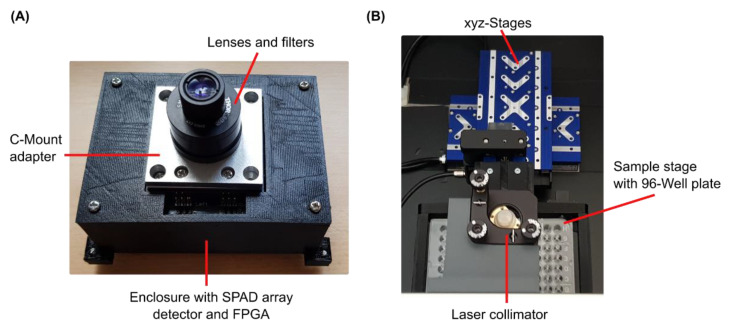
Images of the experimental FL setup: (**A**) SPAD array detector with the lenses and filters attached in a tube housing mounted via a C-mount adapter. (**B**) Top view of the measurement setup with the laser and collimator mounted on top, the optical path of which is directed to a well in the underlying 96-well plate, adjustable in three axes by three stages. The SPAD array detector from (**A**) is located below the illuminated well.

Control and data readout of the SPAD array detector were realized with a FPGA-board. The sample was homogenously illuminated by a collimated pulsed laser diode with a wavelength (λ =450 nm) (laser diode 720-PL450B, Mouser Electronics, Mansfield, TX, USA) filtered by a bandpass filter (BP 445/50, Carl Zeiss Microscopy, Jena, Germany). Laser pulses with a Full Width at Half Maximum (FWHM) = 1.25 ns) and a turn-off time of 0.5 ns were generated by a laser driver (iC-HG (HG8M), iC-Haus, Bodenheim, Germany). The laser driver was also controlled by the FPGA-board. Positive Fresnel lenses (FRP125, FRP0510, Thorlabs, Newton, NJ, USA) were mounted between the sample and SPAD array detector to collect the fluorescence photons. In addition, long-pass filters (#84-737, Edmund Optics, York, UK, cut-off wavelength 475 nm and FELH0500, Thorlabs, Newton, NJ, USA, cut-off wavelength 500 nm) in front of the SPAD array detector were used to filter out the laser beam (Figure 3A). Three stages (*xy*-direction: V-508 PIMag®; *z*-direction: M-122.2DD1, Physik Instrumente, Karlsruhe, Germany) allow to precisely position a 96-well plate with the fluorophore sample solution under the laser beam (Figure 3B).

**Figure 4:**
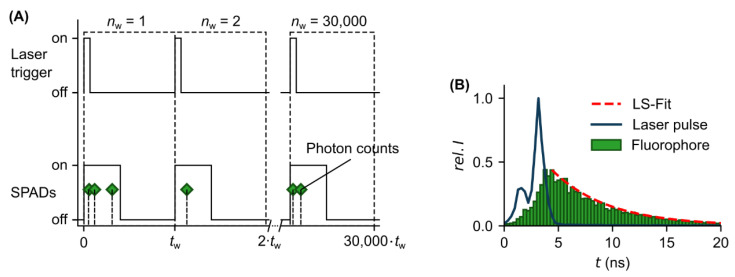
Experimental FL measurement. (**A**) Timing scheme of the measurement procedure. Laser trigger and SPADs were turned on and off during one measurement window. The total number of measurement windows was set to $n_{w}=30,000$. Each detected photon (green markers) during the on phase of the SPADs has a specific arrival time. (**B**) Representative measurement curves for a laser pulse (FWHM=1.25 ns, blue curve) and for the resulting fluorescence signal (histogram of all arrival times, green) detected by the SPAD array detector. The exponential decay of the fluorescence signal was fitted by nonlinear least square (LS) method to determine the FL (red dashed curve).

During one measurement, the laser trigger and SPADs were repeatedly turned on and off (Figure 4A). The SPADs are sensitive for photon detection during the on phase (interval of 1.28 µs). Afterwards, in the off phase (interval of 19.2 µs), the data were read out from the SPAD array detector and the SPADs were reactivated. In total, one measurement was composed of 30,000 measurement windows. Each detected photon during the measurement windows has a specific arrival time. All arrival times are stored in a histogram to extract the FL by nonlinear least square (LS) fitting (Figure 4B).

### 3.2. Fluorescent Dyes

The following fluorophores were used in FL experiments: lucifer yellow (L0259, Merck, Darmstadt, Germany), acriflavine (01673, Merck, Darmstadt, Germany), 2-amino-acridone (06627, Merck, Darmstadt, Germany), and fluorescein (46955, Merck, Darmstadt, Germany). They were solved in deionized water. For 2-amino-acridone and fluorescein, the final concentration in solution was c=15.6 µM. For acriflavine and lucifer yellow, the final concentration in solution was c=3.9 µM.

### 3.3. Monte-Carlo Simulations

**Figure 5:**
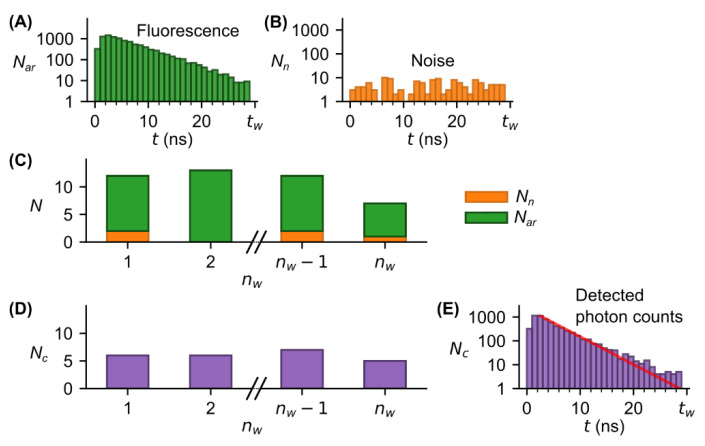
Principle of FL Monte Carlo simulation. (**A**) Distribution of fluorescence photons $N_{ar}$ arriving at the detector. The distribution was obtained by convolving the turn-off function of the laser pulse and the exponential fluorescence decay of the fluorophore. (**B**) Distribution of events that arise from noise sources such as dark counts and scattered light. (**C**) Number of arrived photons per measurement window (randomly Poisson distributed over all measurement windows). (**D**) Number of detected photons per measurement window. Since only one photon can be detected per pixel and measurement window, only the fastest photons are detected. (**E**) Distribution of all detected photons from that the FL is determined by LS fitting.

The experimental FL measurements were validated through a self-written single-photon statistic-based Monte Carlo simulation in Python (Figure 5).

In the FL simulation, for each measurement window an excitation laser pulse induces a Poisson-distributed random number of emitted fluorescence photons. Thereby, the laser pulse characteristics (width and falling edge) were considered (Figure 5A). This number corresponds to the mean photon counts per measurement window $<N_{c}{>}_{w}$ that would be detected without the occurrence of pile-up, which will be referred to here as count rate. Each generated photon has its characteristic arrival time at the SPAD array detector. In addition to the fluorescence photons, random events at the detector from noise sources (dark count rate (DCR), scattered light) were generated (Figure 5B). The level of noise events is in accordance with the photon counts measured by the experimental setup when the excitation laser was off. All generated arrival times and noise events were Poisson distributed among the measurement windows $n_{w}$ (Figure 5C) and then evenly distributed among the pixels of the detector. In our simulation, each pixel can only count one photon per measurement window, which means that not all generated signals were counted (Figure 5D). All counted photons $N_{c}$ are stored in a histogram to determine the FL (Figure 5E).

### 3.4. Statistical Analysis

The FL was determined from the histograms by LS fit with fit function

(1) $$I=I_{0}e^{-\frac{t}{\tau_{\mathrm{fit}}}}\;\;\;,$$

where $I_{0}$ and $\tau_{\mathrm{fit}}$ are the fit parameter for the intensity and the FL, respectively. We calculated the coefficient of variation

(2) $$c_{v}=\frac{\sigma }{\langle \tau_{\mathrm{fit}}\rangle }\;\;,$$

with the arithmetic mean $<\tau_{\mathrm{fit}}>$ and standard deviation σ from multiple measurements (N=100). The obtained $c_{v}$ values at three different sample positions were averaged to one representative mean value for the precision of FL acquisition ${\bar{c}}_{v}$. From the fit parameter $\tau_{\mathrm{fit}}$ and characteristic FL of the fluorophore $\tau_{0}$, we calculated the relative error

(3) $$\delta \tau =\langle \frac{\left|\tau_{\mathrm{fit}}-\tau_{0}\right|}{\tau_{0}}\rangle \;,$$

from multiple measurements (N=100). The obtained δτ values at three different sample positions were averaged to one representative mean value for the accuracy of FL acquisition $\bar{\delta \tau }$. Since $c_{v}$ and δτ approximately followed a log-normal distribution, ${\bar{c}}_{v}$ and $\bar{\delta \tau }$ were calculated as geometric means. Error bars represent (geometrical) the standard error of mean. Data analyses were carried using self-written procedures in Python.

## 4. Results and Discussion

### 4.1. Photon Statistics

**Figure 6:**
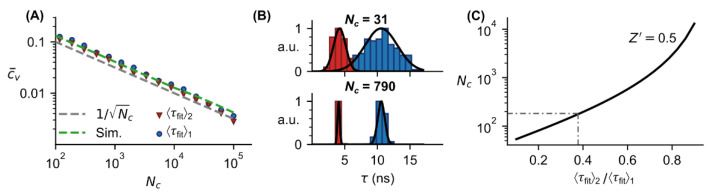
Photon statistics in FL measurements. (**A**) Precision vs. photon counts. Experimental FL measurements of two fluorophores (2-amino-acridone, red and fluorescein, blue) and corresponding Monte Carlo simulations show that the precision ${\bar{c}}_{v}$ depends on the photon counts $N_{c}$ with a $1/\sqrt{N_{c}}$ -dependency independently of the absolute lifetime value as predicted by Poisson statistics. (**B**) Representative distribution of FLs at low $N_{c}$ (upper panel) and high $N_{c}$ (lower panel) for the two fluorophores in (**A**) show that for the larger $N_{c}$ value the standard variation σ is lower while the mean values μ are the same. In experiments, overlapping distributions (**B**, upper panel) are critical for differentiating multiple fluorophores, so higher photon counts are preferable, but in general at cost of longer measurement times. (**C**) Successful differentiation requires a sufficient distance between these two distributions, which can be achieved for $Z^{\prime }=0.5$ (Equation (6)), corresponding to a distance equal to six times the sum of the standard deviations of two fluorophores. Such a distance is described in [29] as an excellent assay, and the number of photons required compared with the ratio of the mean values of two FLs to achieve this is shown here. The grey dashed/dotted line indicates the minimum required photon counts for distinguishing the fluorophores in (**A**,**B**). Error bars in (**A**) are smaller than the markers.

Since the FL determination underlies Poisson statistics, it can only be determined with a certain precision. For this reason, several FL measurements are usually performed in experiments to obtain robust values for the population mean and the standard deviation for the FL of each fluorophore. This is especially important in multiplexing applications aiming to distinguish between different fluorophores (Figure 6) [13]. We carried out FL measurements using the FL measurement setup described in Section 3.1 on two different fluorophores and obtained a mean FL of $\langle \tau_{\mathrm{fit}}\rangle {}_{1}=10.6\;\mathrm{ns}$ and $\langle \tau_{\mathrm{fit}}\rangle {}_{2}=4.1\;\mathrm{ns}$ for 2-amino-acridone and fluorescein, respectively. These values are in good accordance to the literature (2-amino-acridone in water: τ≈10 ns [27], fluorescein in PBS at pH=8: τ=3.99 ns [28]). For the two fluorophores (2-amino-acridone and fluorescein) with the experimentally obtained FLs and Monte Carlo simulated data with a set FL of τ=5 ns, we determined the precision provided by the mean coefficient of variation ${\bar{c}}_{v}$ at different photon counts (Figure 6A). It can be shown that independently from the absolute FL value, ${\bar{c}}_{v}$ depends on the photon counts with $1/\sqrt{N_{c}}$ as expected since photon counting in FL measurements underlies Poisson statistics. Larger photon counts result in lower values for ${\bar{c}}_{v}$ and hence in a more precision FL determination. This coincides with Monte Carlo simulations for τ=5 ns (Figure 6A).

Histograms of FL distributions at two different photon counts for the two fluorophores (2-amino-acridone and fluorescein) show that at relatively low photon counts (corresponding to high $c_{v}$), the distributions overlap while at high photon counts (corresponding to low $c_{v}$), the distributions are clearly separated (Figure 6B). In a high-throughput screening assay, typically the *Z*′ factor is calculated to provide a quantitative value for separation of two distributions and hence for the assay quality [29]

(4) $$Z^{\prime }=1-\frac{\left(3\sigma_{1}+3\sigma_{2}\right)}{\left|\langle \tau_{\mathrm{fit}}\rangle {}_{1}-\langle \tau_{\mathrm{fit}}\rangle {}_{2}\right|}=1-\frac{\left(\langle \tau_{\mathrm{fit}}\rangle {}_{1}+\langle \tau_{\mathrm{fit}}\rangle {}_{2}\right)3c_{v}}{\left|\langle \tau_{\mathrm{fit}}\rangle {}_{1}-\langle \tau_{\mathrm{fit}}\rangle {}_{2}\right|}\;,$$

with the standard deviations $\sigma_{1,2}$ the mean values $\langle \tau_{\mathrm{fit}}\rangle {}_{1,2}$.

From Figure 6A we derived that $c_{v}$ is approximately inversely proportional to the square root of the photon counts [13,21]

(5) $$c_{v}\approx \frac{1}{\sqrt{N_{c}}}\;\;.$$

Inserting Equation (4) in Equation (5) and rearranging to the photon count provides the following relation for $\langle \tau_{\mathrm{fit}}\rangle {}_{1}>\langle \tau_{\mathrm{fit}}\rangle {}_{2}$

(6) $$N_{c}={\left[\frac{3}{\left(1-Z^{\prime }\right)}\left(\frac{1+\frac{\langle \tau_{\mathrm{fit}}\rangle {}_{2}}{\langle \tau_{\mathrm{fit}}\rangle {}_{1}}}{1-\frac{\langle \tau_{\mathrm{fit}}\rangle {}_{2}}{\langle \tau_{\mathrm{fit}}\rangle {}_{1}}}\right)\right]}^{2}\;.$$

For $Z^{\prime }\geq 0.5$, the distributions are sufficiently separated, clarified as an excellent assay according to [29]. For example, with $Z^{\prime }=0.5$, corresponding to six times the sum of the standard deviations of two fluorophores, this provides $N_{c}=184$ for the two fluorophores 2-amino-acridone and fluorescein with a ratio of $\langle \tau_{\mathrm{fit}}\rangle {}_{2}/\langle \tau_{\mathrm{fit}}\rangle {}_{1}=0.39$.

However, most fluorophores have FLs in the range between 1and 4 ns [30], which means that the resulting ratio of $\langle \tau_{\mathrm{fit}}\rangle {}_{2}/\langle \tau_{\mathrm{fit}}\rangle {}_{1}$ is at least 0.25. In this case, based on Equation (6), it follows that more than 100 photon counts are required to achieve $Z^{\prime }=0.5$. To keep the measurement time short the required total photon counts should be acquired as fast as possible. However, shortest measurement time is limited by the pile-up effect.

### 4.2. Pixel Dependency in FL Acquisition

**Figure 7:**
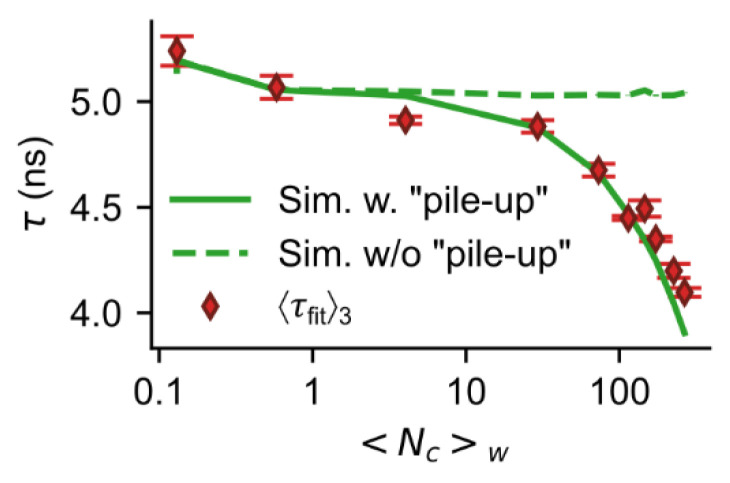
Impact of the detector’s pile-up on the FL. Lifetime vs. count rate $\langle N_{c}\rangle {}_{w}$. Experimental FL measurements (red markers) and Monte Carlo simulations (green curves) for a SPAD array detector show that at lower $\langle N_{c}\rangle {}_{w}$ values the lifetime stays relatively constant. At higher $\langle N_{c}\rangle {}_{w}$ values the impact of detector’s pile-up (explained in Figure 1(Cii)) leads to an increasingly underestimation of the lifetime (difference between Monte Carlo simulation with pile-up (green solid curve) to the respective simulation without pile-up (green dashed curve)).

To evaluate the pile-up effect (see also Section 2), we carried out FL measurements on a fluorophore (acriflavine) while adjusting the fluorescence intensity by using neutral density filters (NDUV01A to NDUV40A, Thorlabs, USA) in front of the SPAD array detector. This allowed us to determine the mean FL at different mean photon counts per measurement window (Figure 7).

At low count rates ($\langle N_{c}\rangle {}_{w}<10$) the determined FL remains roughly constant (${\langle \tau_{\mathrm{fit}}\rangle }_{3}\approx 5\;\mathrm{ns}$). Beyond that, with increasing count rates the determined FL deviates significantly from the FL at low count rates, which is confirmed by the Monte Carlo simulation (green solid curve, Figure 7). If we carry out the simulation without taking the pile-up into account, the resulting FL stays constant independently of the count rate (green dashed curve, Figure 7). It must be mentioned that in the simulation we assumed that all pixels see the same count rate. However, this was not the case in the FL experiments due to the Gaussian nature of the laser’s beam profile. Therefore, only SPAD pixels illuminated with at least 50% of max intensity (photon counts) were considered (i.e., 233 of the 2 × 192 active SPAD pixels).

**Figure 8:**
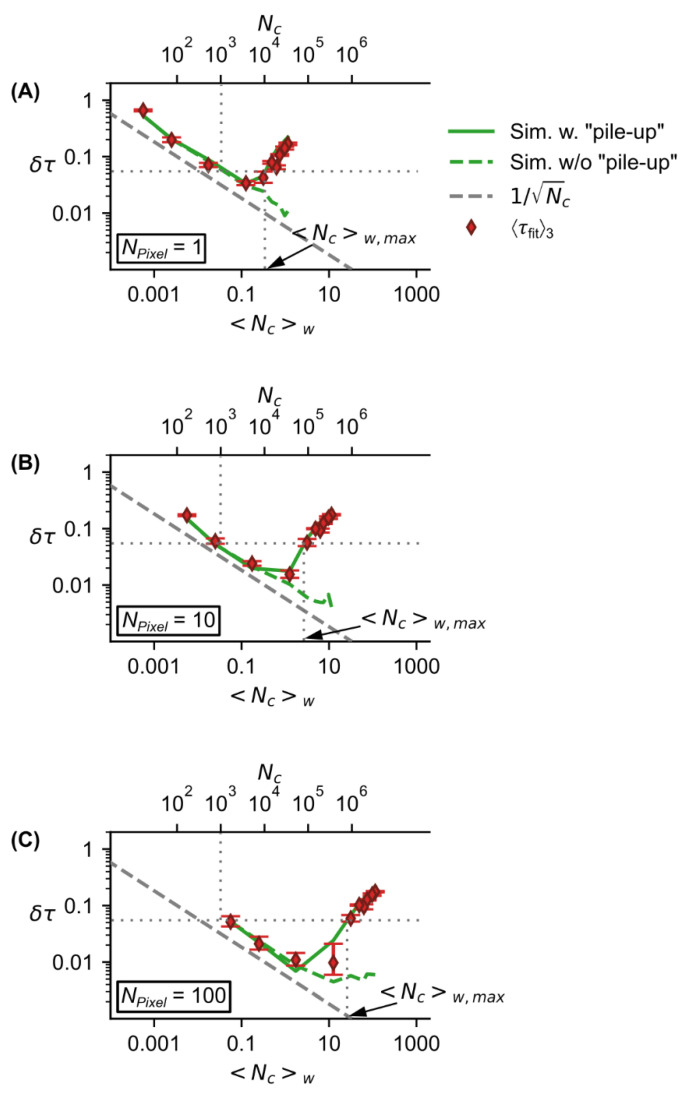
Impact of the detector’s pile-up on relative accuracy of determined FL. Relative accuracy δτ of the measured lifetime τ vs. count rate $\langle N_{c}\rangle {}_{w}$ for different number of the SPAD array detector’s active pixels (**A**, 1 pixel; **B**, 10 pixels; **C**, 100 pixels). At a relative accuracy of δτ=5.5% which corresponds to a photon counts of $N_{c}=1000$ the maximum count rate $\langle N_{c}\rangle {}_{w,\max}$ is larger for higher number of pixels (**A**–**C**).

To show the pile-up’s influence independently of the absolute FL and considering the number of pixels, we determined the relative accuracy δτ (Equation (3)) as a dimensionless value (Figure 8).

Similar to the dependency of the precision (Figure 6A), the relative accuracy initially decreases with increasing photon counts (Figure 8A–C, upper *x*-axis) according to $1/\sqrt{N_{c}}$ (Figure 8A–C, grey dashed curve), while keeping the number of measurement windows constant at $n_{w}=30,000$. In contrast to the precision, the relative accuracy starts to increase again at a specific level of total photon counts. This level corresponds to a certain count rate

(7) $$<N_{c}{>}_{w}=\frac{N_{c}}{n_{w}}\;\;\;.$$

The different dependency of the precision and relative accuracy on the count rate is to be expected, since the precision is provided by the coefficient of variation which solely depends on the photon counts (Equation (5)).

The pile-up is negligible when the accuracy of the FL determination depends mainly on the photon counts (in the range where the green solid curves in Figure 8A–C follow the gray dashed curve).

**Figure 9:**
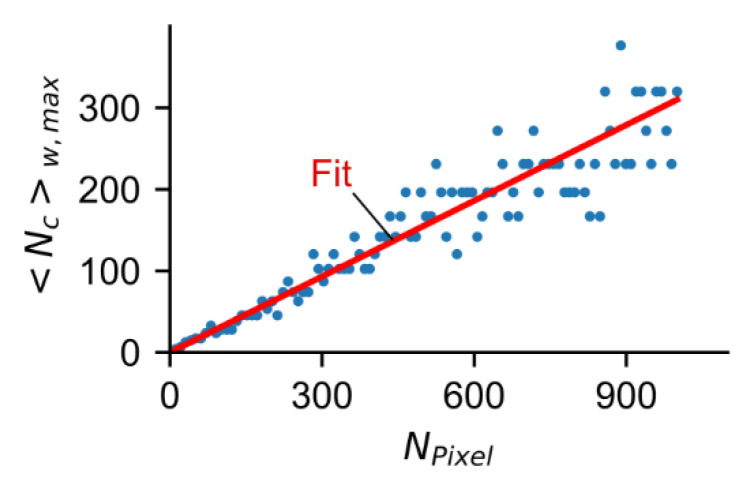
$\langle N_{c}\rangle {}_{w,\max}$ vs. $N_{\mathrm{Pixel}}$ determined from Monte Carlo simulations show a linear correlation.

From Figure 8A–C, we determined for $N_{c}=1000$ photon counts, the relative accuracy is 5.5%. For this level of relative accuracy there is a maximum count rate $\langle N_{c}\rangle {}_{w,\max}$ before the pile-up deteriorates the relative accuracy (back to higher values) (Figure 8A–C). The $\langle N_{c}\rangle {}_{w,\max}$ values depend on the number of pixels (Figure 8A–C). To obtain a large number of $\langle N_{c}\rangle {}_{w,\max}$ values for a large number of pixels in the range 1–1000; we carried out Monte Carlo simulations and the results show that there is a linear correlation between $\langle N_{c}\rangle {}_{w,\max}$ and $N_{\mathrm{Pixel}}$ (Figure 9).

From the line fit (Figure 9, red line), we obtained a maximum count rate per pixel $<N_{c}{>}_{w,\max}/N_{\mathrm{Pixel}}=0.31$. Note that this parameter is valid for the specific example of a relative accuracy of 5.5%. In the case of higher accuracy (lower values of accuracy) the maximum count rate per pixel would be lower. Assuming one pixel, this value is comparable to the count rate of an ideal PMT of 0.37 [17]. In TCSPC, the maximum count rate is even chosen slightly lower in the range of 0.1 to 0.2 to ensure that no pile-up occurs [20]. Comparing the maximum count rate of the SPAD array detector and PMT detector, for fast FL determination the SPAD array detector is superior even with a small number of pixels (larger than two).

### 4.3. Total Measurement Time and Data Rate

In order to perform a fast FL determination, it is necessary to detect as many photons in the shortest possible time. However, as discussed in Section 4.2, this is not trivial since only a maximum mean count rate is possible before pile-up affects the determined FL. Another factor influencing the measurement time is the duration of a single measurement window, which should also be short as possible, but so long that almost all emitted photons arrive on the detector within the measurement window. Otherwise, “slow” photons can be detected in the subsequent measurement window as “fast” photons.

Many standard fluorophores, such as those used in this study, display a single exponential decay

(8) $$I=I_{0}e^{-\frac{t}{\tau }}\;\;,$$

with the Intensity $I_{0}$ at time t=0.

The percentage of emitted photons from the fluorophore after a laser pulse excitation can be extracted from

(9) $$P=\frac{N_{\Delta t_{w}}}{N_{\mathrm{All}}}=\frac{\int_{0}^{\Delta t_{w}}I}{\int_{0}^{\infty }I}=\frac{{\left[I_{0}\left(-\tau \right)e^{-\frac{t}{\tau }}\right]}_{0}^{\Delta t_{w}}}{{\left[I_{0}\left(-\tau \right)e^{-\frac{t}{\tau }}\right]}_{0}^{\infty }}=1-e^{-\frac{\Delta t_{w}}{\tau }}\;\;.$$

**Figure 10:**
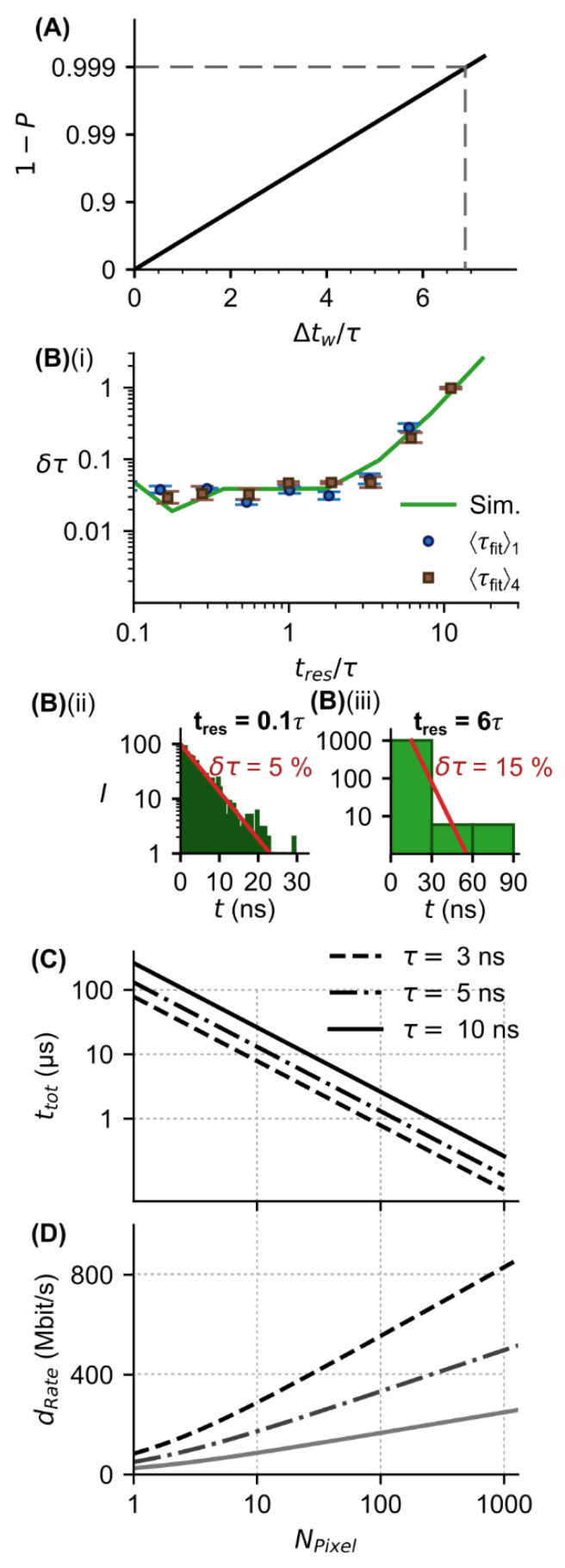
SPAD array detector: impact of the number of pixels on the total measurement time and data rate. (**A**) Proportion of emitted photons that arrive at the detector within the measurement window duration $t_{w}$ (normalized by the lifetime). A total of 99.9% of emitted photons arrive at the detector within $t_{w}/\tau =7$. (**Bi**) Relative accuracy of lifetime acquisition from fitting the histograms depends on the binning, i.e., time resolution of the detector (for $N_{c}=1000$ ). Experimental measurements (blue and brown markers) and Monte Carlo simulations (green curve) show that with decreasing time resolution, δτ values are at a constant level until approx. $t_{\mathrm{res}}=2\tau$ and then becoming larger, reaching δτ=10% at $t_{\mathrm{res}}=4\tau$ due to the increasing impact of noise (e.g., DCR, stray light). Representative histograms for simulated data show that at a low time resolution of $t_{\mathrm{res}}=6\tau$ (**Bii**) almost all photon counts from the fluorescence signal are in the first bin. The other bins only contain photon counts from noise source, resulting in a higher 〈Δτ/τ〉 value (lower accuracy) after fitting compared with a high time resolution of $t_{\mathrm{res}}=0.1\tau$ (**Biii**). (**C**) Total measurement time vs. number of pixels for different lifetimes. With increasing lifetime τ the total measurement time $t_{\mathrm{tot}}$ increases according to Equation (10), since $t_{w}$ must be longer if almost all emitted photons should arrive within $t_{w}$. With increasing number of pixels $N_{\mathrm{Pixel}}$ the total measurement time $t_{\mathrm{tot}}$ decreases according to Equation (11), since higher count rates can be tolerated (Figure 9). (**D**) Relationship between estimated required data rate of SPAD array detector with on-chip digital signal processing (CMOS device) and number of pixels for different lifetimes (time resolution $t_{\mathrm{res}}$ was set to 4τ ). As the number of pixels increases, the data rate increases according to Equation (12).

According to this function, for a proportion of 99.9% emitted photons a minimum duration of the measurement window is $\Delta t_{w}=7\cdot \tau$ (Figure 10A). It is unlikely that photons of the remaining 0.1% fraction will be detected after this time, so the next measurement window can start right after that.

Next to the duration of a measurement window, an important factor that must be considered regarding the total measurement time is the data rate of the chip containing the SPAD array detector and evaluation circuitry. This is, among other factors, influenced by the chip’s time-resolution since higher time-resolution means more data in a shorter time. To evaluate how the accuracy of FL determination is affected by the time-resolution, we carried out FL measurements on two different fluorophores with the SPAD array detector’s time-resolution of 312.5 ps. We obtained for 2-amino-acridone and for lucifer yellow a mean FL of $\langle \tau_{\mathrm{fit}}\rangle {}_{1}=10.6\;\mathrm{ns}$ and $\langle \tau_{\mathrm{fit}}\rangle {}_{4}=5.7\;\mathrm{ns}$, respectively. These values are in good accordance to the literature (2-amino-acridone in water: τ≈10 ns [27], lucifer yellow: τ=5.29 ns [31]). In order to obtain lower time resolutions, the width of the time resolution was then resized afterwards during the evaluation of the measurement results (Figure 10(Bi)).

It can be shown that regardless of the absolute FL, the accuracy is approximately constant while the time resolution is smaller than two times the FL ($t_{\mathrm{res}}<2\tau$) (Figure 10(Bi)). For the line fit at least two bins with photon counts representing the single-exponential decay are required. If the time resolution is much larger than the FL, then most of the detected photon counts within the second bin result from noise (e.g., DCR, scattered light) and the relative accuracy decreases (Figure 10(Bi)). To emphasize these findings, in Figure 10(Bii,Biii) two representative histograms for simulated data at a relatively high time resolution of $t_{\mathrm{res}}=0.1\tau$, where photon counts are widely distributed over the bins (resulting in high accuracy of FL) and at a low time resolution of $t_{\mathrm{res}}=6\tau$, where almost all photon counts are in the first bin (resulting in low accuracy of FL), are shown. With a maximum FL accuracy of 10%, the required minimum time resolution is $t_{\mathrm{res}}=4\tau$ (Figure 10(Bi)). By increasing photon counts and reducing experimental noise, this limit can be increased (data not shown).

In conclusion, these results show that the time resolution does not necessarily need to be in the ~ps range to determine the FL with a sufficient accuracy. Since more data are generated at higher time resolution, reducing the required time resolution speeds up the chip’s data rate.

In the following section, we discuss the pros and cons of using pixelated array detectors in the context of measurement time and data rate. The total measurement time $t_{\mathrm{tot}}$ is determined by the number of measurement windows $n_{w}$ times the duration of a measurement window $\Delta t_{w}$. The number of measurement windows is provided by the ratio of the photon counts $N_{c}$ and the count rate $<N_{c}{>}_{w}$

(10) $$t_{\mathrm{tot}}=n_{w}\cdot \Delta t_{w}=\frac{N_{c}}{<N_{c}{>}_{w}}\cdot \Delta t_{w}\;\;.$$

As an example, we determined the total measurement time $t_{\mathrm{tot}}\left(\tau ,N_{\mathrm{Pixel}}\right)$ in dependency of the lifetime and the number of pixels by inserting $\Delta t_{w}=7\tau$ (based on the results in Figure 10A) and $<N_{c}{>}_{w}=0.31\cdot N_{\mathrm{Pixel}}$ (based on the results in Figure 9) in Equation (10). It then follows

(11) $$t_{\mathrm{tot}}=\frac{7000\cdot \tau }{0.31\cdot N_{\mathrm{Pixel}}}\;\;.\;$$

In Figure 10C, it is shown that for different FLs, the total measurement time decreases with an increasing number of pixels. For a given number of pixels, the total measurement time is shorter the smaller FL is, since the minimum duration of the measurement window is shorter (Figure 10A). Starting at about 30 pixels, the measurement time (for τ≤10 ns) is less than 10 µs, and starting at 300 pixels, a measurement time of less than 1 µs is required to acquire 1000 photon counts and thus determine the FL with a precision and accuracy of 4% and of 5.5%, respectively. The total measurement time cannot be shorter than the FL of the analyzed fluorophore. Another limiting factor for the total measurement time is the fluorophore concentration, which determines the number of detectable photons per laser pulse.

To determine the data rate, we used a time resolution of $t_{\mathrm{res}}=4\tau$ according to the results in Figure 10(Bi) and assumed that the photon counts from the SPAD array detector’s pixels were summed to one photon count value after each measurement window and forwarded to a FPGA for further data processing. Note that in this case the pixel information and the imaging capabilities of the SPAD array detector were lost. We obtain following Equation for the data rate

(12) $$d_{\mathrm{rate}}=\frac{{log}_{2}\left(N_{\mathrm{Pixel}}+1\right)}{t_{\mathrm{res}}}=\frac{{log}_{2}\left(N_{\mathrm{Pixel}}+1\right)}{4\cdot \tau }\;\;\;.$$

As expected, Figure 10D shows that the data rate increases with increasing number of pixels. Shorter FLs require a higher data rate since the required time resolution is higher. However, the data rate remains below 1 Gbit/s even with a number of pixels of 10^3^. These rates can be transferred with modern digital circuits (for comparison USB 3.0: 5 Gbit/s [32]) and evaluated for example using an FPGA. In this case, the data rate does not limit the FL measurement time.

## 5. Conclusions

In this study, we reported the advantages and limitations of using SPAD array detectors to precisely and accurately measure FLs. We showed that the precision underlies Poisson statistics and depends on the photon counts $N_{c}$ with a $1/\sqrt{N_{c}}$-dependence, independently of the absolute FL value. We derived an Equation that provides the required photon counts in dependency of an assay quality factor (*Z*′ value) in order to differentiate two fluorophores with certain FLs.

Furthermore, we investigated the correlation between the FL accuracy and the count rate for different detector’s number of pixels. It can be shown that for a higher number of pixels the count rate can be higher before the detector’s pile-up negatively impacts the accuracy. We obtained a maximum count rate per pixel of 0.31. It follows that with a higher number of pixels, the statistically required photons for a certain precision and accuracy can be detected in a shorter total measurement time, emphasizing the main advantage of using pixelated SPAD array detectors instead of “single-pixel”, e.g., PMT detectors. For simplification, we assumed that photon arrival events at the detector are evenly distributed among the pixels. In typical experimental setups, the irradiation of the detector and therefore the photon distribution is not homogenous due to a Gaussian beam profile. This in turn impacts the value of the maximum count rate for each pixel since for the inner pixels the photon rate is typically higher than for the outer pixels. However, beam-shaping optics can be used to optimize the irradiation profile on the detector, but at the cost of a more complex setup.

The total measurement time results from the cyclic repetition of the measurement window, in which excitation and detection take place once each. The length of the measurement window should be chosen so that there is a probability of detecting almost all emitted photons that arrive at the detector. We provided an Equation for the minimum duration of the measurement window provided by 7τ. We showed that FLs <10 ns with a precision of 3% and an accuracy of 5.5% can be determined with a SPAD array detector with 30 and 300 SPAD pixels within a measurement time less than 10 µs and less than 1 µs, respectively. SPAD array detectors with a high number of pixels have already been developed, e.g., a 512 × 512 SPAD array [33].

With a high number of pixels, the data rate in bits/s is correspondingly high. One approach to reduce the data rate is to lower the time resolution at which the photons are detected. We showed that even with a time resolution of $t_{\mathrm{res}}=4\tau$, FL accuracy of 10% is reached. In this case, even with 1000 pixels and FLs greater than 3 ns, data rates remain below 1 Gbit/s. This is achievable with modern digital circuits and makes continuous determination of FLs conceivable, e.g., for high-speed applications such as flow cytometry with measurement times per cell in the µs range. The findings reported in our study can serve as a foundation for the development of dedicated high-speed SPAD-based array detectors.

## Data Availability

Experimental data are available upon request.
